# Comparison of Multilocus Variable-Number Tandem-Repeat Analysis and Whole-Genome Sequencing for Investigation of Clostridium difficile Transmission

**DOI:** 10.1128/JCM.01095-13

**Published:** 2013-12

**Authors:** D. W. Eyre, W. N. Fawley, E. L. Best, D. Griffiths, N. E. Stoesser, D. W. Crook, T. E. A. Peto, A. S. Walker, M. H. Wilcox

**Affiliations:** NIHR Oxford Biomedical Research Centre, University of Oxford, John Radcliffe Hospital, Oxford, United Kingdoma; Leeds Teaching Hospitals NHS Trust, Leeds, United Kingdomb; Leeds Institute of Biomedical & Clinical Sciences, University of Leeds, Leeds, United Kingdomc

## Abstract

No study to date has compared multilocus variable-number tandem-repeat analysis (MLVA) and whole-genome sequencing (WGS) in an investigation of the transmission of Clostridium difficile infection. Isolates from 61 adults with ongoing and/or recurrent C. difficile infections and 17 asymptomatic carriage episodes in children (201 samples), as well as from 61 suspected outbreaks affecting 2 to 41 patients in 31 hospitals in the United Kingdom (300 samples), underwent 7-locus MLVA and WGS in parallel. When the first and last samples from the same individual taken for a median (interquartile range [IQR]) of 63 days (43 to 105 days) apart were compared, the estimated rates of the evolution of single nucleotide variants (SNVs), summed tandem-repeat differences (STRDs), and locus variants (LVs) were 0.79 (95% confidence interval [CI], 0.00 to 1.75), 1.63 (95% CI, 0.00 to 3.59), and 1.21 (95% CI, 0.00 to 2.67)/called genome/year, respectively. Differences of >2 SNVs and >10 STRDs have been used to exclude direct case-to-case transmission. With the first serial sample per individual being used to assess discriminatory power, across all pairs of samples sharing a PCR ribotype, 192/283 (68%) differed by >10 STRDs and 217/283 (77%) by >2 SNVs. Among all pairs of cases from the same suspected outbreak, 1,190/1,488 (80%) pairs had concordant results using >2 SNVs and >10 STRDs to exclude transmission. For the discordant pairs, 229 (15%) had ≥2 SNVs but ≤10 STRDs, and 69 (5%) had ≤2 SNVs but ≥10 STRDs. Discordant pairs had higher numbers of LVs than concordant pairs, supporting the more diverse measure in each type of discordant pair. Conclusions on whether the potential outbreaks were confirmed were concordant in 58/61 (95%) investigations. Overall findings using MLVA and WGS were very similar despite the fact that they analyzed different parts of the bacterial genome. With improvements in WGS technology, it is likely that MLVA locus data will be available from WGS in the near future.

## INTRODUCTION

Molecular typing significantly enhances Clostridium difficile outbreak investigation and transmission studies, as well as informing surveillance efforts (1, 2). PCR ribotyping and pulsed-field gel electrophoresis (PFGE) are currently the most frequently used typing techniques in Europe and North America, respectively (1). However, multilocus variable-number tandem-repeat analysis (MLVA) (3–5) and whole-genome sequencing (WGS) of C. difficile (6, 7) both offer increased discrimination over other typing schemes.

MLVA exploits variations in the copy number at ≥7 tandem-repeat loci to distinguish isolates. Differences between the isolates are reported in terms of the number of loci at which they differ (locus variants [LVs]) and the sum of the absolute differences in copy number at each locus (summed tandem-repeat difference [STRD]). WGS can be used to compare single nucleotide variants (SNVs) between isolates across the nonrepetitive core genome, which accounts for ∼80% of the 4.3 million-base pair C. difficile strain 630 reference genome (6–8). MLVA and WGS analyze completely different parts of the C. difficile genome and reflect different evolutionary processes; they may therefore have differing performances as tools for C. difficile surveillance and epidemiology.

An ideal typing scheme is reproducible and able to discriminate efficiently between isolates from a given population of interest. In contrast to categorical typing schemes, like ribotyping and PFGE, WGS and MLVA continuously grade the relatedness of isolates. This has the potential to allow for the reconstruction of transmission chains by tracking the accumulation of changes over time and in different hosts. An ideal metric changes monotonically with time, without significant back mutation. To follow infections between successive hosts, the rate of change should be low enough that transmitted isolates are clearly related but sufficiently high relative to the rate of transmission and within-host diversity for changes to accumulate in successive hosts that allow for the transmission chains to be ordered. The extent of within-host variation during a single infection should also be low compared with the population diversity.

With MLVA, reproducibility has been assessed by repeated culture of the same strain and serial subculture, and ≤1 STRD was observed (1 strain was cultured 5 times, and 5 strains were serially subcultured 10, 11, 11, 11, and 30 times) across 2 studies (9, 10). One strain had evidence of expansion and reversion of a single locus by 1 repeat during subculture (9). Differences of up to 1 repeat unit per locus were seen in a comparison of agarose gels and capillary sequencing, highlighting the contribution of the assay method to the detection of variations (11). Within-host diversity was assessed by typing five colonies from the same specimen, and 34/39 (87%) colonies had ≤2 STRDs. The extent to which the diversity observed in the remaining five cases (5 to 24 STRDs) represents a mixed infection with another strain is unclear, as all isolates were from the highly prevalent epidemic C. difficile ribotype 027 (11). Across two studies, in 13 serially sampled patients with samples 2 days to 8 months apart, up to 3 STRDs were observed, with 2 STRDs after 2 days in 1 patient and 3 STRDs after 20 days in another (3, 10). These data have led to the consensus that isolates ≤2 STRDs apart should be regarded as indistinguishable. The clustering of cases 3 to 10 STRDs apart within diverse strain collections (3, 12) has led to such cases being regarded as closely related, consistent with local outbreaks (5, 13, 14). However, a recent analysis of 117 recurrent episodes using MLVA highlights the uncertainty surrounding how many STRDs are compatible in the same infection over time, with 16 recurrent episodes having between 3 and 9 STRDs (15). Extensions to the original MLVA scheme using 14 or 15 loci have been proposed as alternatives to the combination that is typically used of PCR ribotyping followed by 7-locus MLVA (16, 17).

With WGS, the appropriate data processing pipelines result in ∼1 false SNV identified per 100 genomes sequenced (based on 66 genomes that were sequenced between 2 and 8 times) (7). The rates of C. difficile evolution estimated from 91 serially sampled patients and a global collection of ribotype 027 strains are similar, at ∼1 to 2 SNVs per genome per year (7, 18). From serially sampled patients and five samples with 12 colonies sequenced from each patient, the extent of within-host diversity is typically ≤1 SNV (7, 19).

To compare the utility of MLVA versus WGS in outbreak investigations, we first investigated a comprehensive set of serially sampled adults with ongoing or recurrent C. difficile infections (CDI) and asymptomatic children (aged <2 years) using the two methods. The performance of MLVA versus that of WGS was then compared across 61 potential CDI outbreaks (5).

## MATERIALS AND METHODS

### Patients/participants and samples.

Serially sampled adults with ongoing or recurrent CDIs were identified from a collection of all hospital and community samples from the county of Oxfordshire, United Kingdom, between September 2006 and July 2010, which was previously described in detail (2, 20). Toxin enzyme immunoassay (EIA)-positive samples underwent culture and multilocus sequence typing (MLST) (21). Three hundred twenty-five patients were identified with the same sequence type on multiple samples. Sixty-one of these patients were selected at random for further study using MLVA and WGS (148 samples; 40 patients had 2 samples, 16 had 3 samples, and 5 had 4 samples).

Samples were also obtained from an ongoing study of C. difficile carriage in asymptomatic children aged <2 years (22) to assess if this group, who were without symptoms and not subject to antibiotic treatment, had different rates of C. difficile evolution and within-host diversity. Following parental consent, longitudinal samples were obtained at approximately monthly intervals for up to 9 months. Sixteen children carrying the same C. difficile ribotype for >1 month were included; one child had two episodes of carriage with two different ribotypes, providing a total of 17 participant-ribotype carriage episodes for study using MLVA and WGS (eight lasting 2 months, three lasting 3 months, one lasting 4 months, four lasting 5 months, and one lasting 6 months).

Sixty-one potential CDI outbreaks involving 300 patients from 31 hospitals in the United Kingdom were investigated using MLVA and WGS. These samples were submitted to the C. difficile Ribotyping Network for England and Northern Ireland (CDRN) between June 2007 and July 2011 by hospitals in which there was clinical suspicion of an outbreak based on a clustering of cases in a particular time and space. Isolates from these clusters of cases found to share a ribotype underwent analysis with MLVA, as presented previously (5), and underwent whole-genome sequencing in this study.

### MLVA.

MLVA was performed as described previously (5). Briefly, seven regions within the C. difficile genome known to contain short tandem repeats, designated A6, B7, C6, E7, F3, G8, and H9, were targeted. Three separate PCR duplexes (A6-G8, B7-F3, and C6-E7) and one single PCR (H9) were used. The forward PCR primers for all loci were labeled with either 6-carboxyfluorescein (FAM), PET, 2′-chloro-5′-flouro-7′,8′-fused phenyl-1,4-dichloro-6-carboxyfluorescein (NED), or 2′-chloro-7′-phenyl-1,4-dichloro-6-carboxyfluorescein (VIC). Repeats were amplified using a single protocol (9). PCR fragments were analyzed using multicolored capillary electrophoresis on an ABI3130xl genetic analyzer, with GeneScan 600 LIZ as an internal marker. Fragment sizes were determined with the GeneMapper software (Applied Biosystems, Life Technologies, Grand Island, NY). The MLVA amplification conditions were adjusted for PCR ribotypes 078 and 017 due to potential sequence mismatches in the primer annealing sites for the loci A6, B7, C6, and G8 (A6 is absent in ribotype 078) (23). For ribotypes with potential mismatches in the primer annealing sites, and for those not previously evaluated using MLVA, selected isolates were sequenced to verify the accuracy of assignments of repeat numbers (data not shown).

### Whole-genome sequencing.

DNA for sequencing was extracted using a commercial kit (QIAamp [Qiagen, Hilden, Germany] or QuickGene [Fujifilm, Tokyo, Japan]). Samples were prepared for sequencing using standard Illumina (San Diego, CA) and adapted protocols. Pools of 96 samples were sequenced at the Wellcome Trust Centre for Human Genetics, Oxford, United Kingdom, on the Illumina HiSeq 2000 platform, generating 100-bp reads. Properly paired sequence reads were mapped using Stampy version 1.0.17 (without Burrows-Wheeler Aligner premapping, using an expected substitution rate of 0.01) (24) to the C. difficile 630 reference genome (GenBank accession no. AM180355.1) (8).

Single nucleotide variants (SNVs) were identified across all mapped nonrepetitive sites using SAMtools (version 0.1.18) (25) mpileup with the extended base-alignment quality flag, after parameter tuning was performed based on bacterial sequences (options -E -M0 –Q25 -q30 -m2 -D -S; other values were defaults). Repetitive regions were identified using BLAST (26) searches of the reference genome using fragments of the same genome. GATK version 1.4.21 (27) was used to create variant call format (VCF) files of the annotated variant sites. We used only SNVs that were supported by ≥5 reads, including one in each direction. A consensus of ≥90% of high-quality bases (Phred scaled quality, ≥25) was also required to support an SNV, and calls had to be homozygous under a diploid model. The calls required the proportion of bases of quality of ≥25 in reads spanning the site of interest to be ≥0.35. A median (interquartile range [IQR]) of 84.0% (83.8% to 84.8%) of the C. difficile 630 reference genome was called across all sequenced isolates.

Adjustment for any clustering of SNVs that was suggestive of recombination was undertaken using the method described in Golubchik et al. (28). The parameter values for the recombination adjustment were obtained from Didelot et al. (7). In cases in which the parameters were not available for a specific lineage, they were obtained from the genetically closest lineage.

### Analysis.

Models were fitted to data from the first and last samples from serially sampled adults and children to evaluate the relationship between variations in SNVs, STRDs, LVs (*s*), and time (*t*). SNVs, STRDs, and LVs were assumed to arise as the combination of a time-dependent Poisson process, representing evolution (at a rate of μ events per unit of time), and a time-independent Poisson process (θ) representing within-host diversity and assay variation (29): *s* ∼ *Pois*(μ*t* + θ).

To confirm evidence for detectable evolution, a separate model was fitted without the time-dependent term (μ*t*) and compared. We also investigated evidence for an excess of samples with zero variants, consistent with a subset of recurrences arising from spores with arrested evolution. In this zero-inflated Poisson model, samples had a time-independent probability of having zero variants, and otherwise, variants were assumed to arise as in the main model.

Models were fitted by the maximum-likelihood method using numerical optimization in R version 2.15.3 (see http://www.r-project.org), with the rates of evolution and within-host diversity and assay variation constrained to be positive. Model comparisons were undertaken using the Akaike information criterion (AIC). Confidence intervals were generated by parametric bootstrap analysis using 1,000 iterations. In a sensitivity analysis, estimates were recalculated after excluding a single outlying pair of samples separated by 561 days.

### Nucleotide sequence accession number.

The sequences reported in this paper have been deposited in the European Nucleotide Archive Sequence Read Archive under study accession no. PRJEB4640 and are available at http://www.ebi.ac.uk/ena/data/view/PRJEB4640.

## RESULTS

### MLVA and WGS within a host over time.

The first and last samples obtained from 61 adult CDI cases with ongoing or recurrent infection and 17 pediatric C. difficile carriage episodes were compared using MLVA and WGS. One CDI case with a probable ribotype 027 reinfection was excluded (the last sample had 16 SNVs, 6 LVs, and 31 STRDs different from the first). The remaining 77 sample pairs were obtained a median (IQR) (range) of 63 (43 to 105 days) (2 to 561 days) days apart.

**Fig 1:**
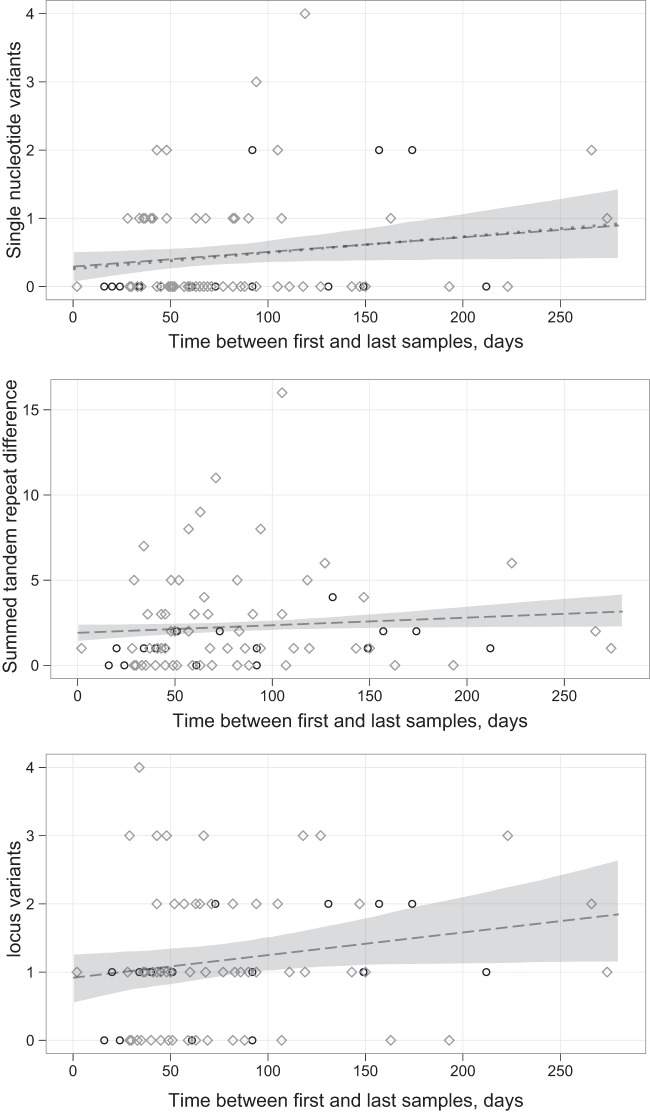
Numbers of single nucleotide variants, summed tandem-repeat differences, and locus variants arising over time. Shown are samples from 60 patients with CDI (gray diamonds) and 17 infant asymptomatic carriage episodes (black circles). The dashed line shows the fitted rate of evolution, and the shaded area represents the 95% confidence interval for the fitted line. A single pair of samples separated by 561 days with 0 SNVs, 4 STRDs, and 3 LVs was not plotted for ease of visualization. In the top panel, the fitted rate of evolution using the same model and data from a previously published collection (19) of 145 serially sampled patients is shown with a dotted lined for comparison.

There was evidence of evolution in the numbers of SNVs, STRDs, and LVs over time; models that included an evolution term showed a better fit to the data than those without (Table 1). The difference in model fit with and without evolution was most marked for SNVs. The estimated rates of evolution in the numbers of SNVs, STRDs and LVs over time were 0.79 (95% CI, 0.00 to 1.75), 1.63 (95% CI, 0.00 to 3.59), and 1.21 (95% CI, 0.00 to 2.67)/called genome/year, respectively (Fig. 1). By all three measures, there was evidence of within-host diversity or intrinsic assay variation, as demonstrated by a significant number of differences estimated at a *t* value of 0 (intercept), namely, 0.29 SNVs (95% CI, 0.05 to 0.53), 1.91 STRDs (95% CI, 1.37 to 2.40), and 0.92 LVs (95% CI, 0.54 to 1.28). Relative to the rates of evolution, there was the least amount of within-host diversity using SNVs, with which it took 0.37 years to generate the same amount of diversity by evolution as observed at baseline, compared to 1.17 years with STRDs and 0.76 years with LVs (Table 1).

**Table 1:** Rates of evolution and baseline diversity^*a*^

Variant types	Model description	Evolution per yr (no. [95% CI])	Within-host diversity (no. [95% CI])	Estimated proportion with fixed zero diversity (no. [95% CI])	Model AIC^*b*^	Time to evolution equaling within-host diversity (yr)
Single nucleotide variants	Within-host diversity only		1.00 (1.00–1.22)		175.6	
	Evolution and within-host diversity	0.79 (0.00–1.75)	0.29 (0.05–0.53)		149.7	0.37
	Evolution, within-host diversity, and proportion with zero diversity	2.02 (0.00–4.15)	0.36 (0.00–0.88)	0.41 (0.00–0.62)	146.6	0.18
Summed tandem-repeat differences	Within-host diversity only		2.30 (1.97–2.64)		383.5	
	Evolution and within-host diversity	1.63 (0.00–3.59)	1.91 (1.37–2.40)		382.7	1.17
	Evolution, within-host diversity, and proportion with zero diversity	0.66 (0.00–3.28)	2.76 (2.03–3.33)	0.22 (0.11–0.32)	363.2	4.21
Locus variants	Within-host diversity only		1.21 (1.00–1.47)		211.5	
	Evolution and within-host diversity	1.21 (0.00–2.67)	0.92 (0.54–1.28)		209.9	0.76
	Evolution, within-host diversity, and proportion with zero diversity	1.21 (0.00–2.77)	0.92 (0.54–1.34)	0.00 (0.00–0.18)	211.9	0.76

a Three models were fitted for each of the following: single nucleotide variants, summed tandem-repeat differences, and locus variants. The within-host-diversity-only model allows for variants to arise as a time-independent Poisson process. The evolution and within-host-diversity model allows for variants to arise and a combination of a time-dependent Poisson process to represent evolution and a time-independent Poisson process to represent within-host diversity or assay variation. The final model, evolution, within-host diversity, and proportion with zero diversity, allows for a proportion of pairs to have arrested evolution such that the proportion of pairs with zero variants is inflated; otherwise, variants arise as in the evolution and within-host-diversity model.

b AIC, Akaike information criterion. Lower values indicate a better model fit. Values can be compared within the SNV, STRD, and LV sections but not across sections.

The broad confidence intervals, including zero for evolutionary rates in all three measures, likely reflect the limited sample size that underwent both MLVA and WGS. For example, fitting the same model to a previously reported set of WGS data from 145 serially sampled cases (which includes the current adult cases) (19) estimates a rate of evolution of 0.86 (95% CI, 0.23 to 1.50) SNVs/called genome/year (Fig. 1A, dotted line). In a sensitivity analysis, when the models were fitted without an outlying pair of samples separated by 561 days and 0 SNVs, 4 STRDs, and 3 LVs, only the estimated rate of evolution for SNVs changed appreciably, to 1.30 (95% CI, 0.10 to 2.48) SNVs/called genome/year. For SNVs and LVs, there was no evidence to support different rates of evolution and within-host diversity for symptomatic adults and asymptomatic children (AIC for the combined SNV model, 149.7, versus separate adults and children model, 152.9, and for the LV models, 209.9 versus 214.4 [with lower values indicating a better model fit], respectively). For STRDs, including separate rates for adults and children improved the model fit (AIC, 383.7 for the combined model versus 373.5 for the separate model), with higher estimated rates of evolution in children (adults, 1.24 [95% CI, 0.00 to 3.67] versus children, 3.09 [95% CI, 0.00 to 6.29] STRDs/called genome/year), but lower rates of within-host diversity (2.31 [95% CI, 1.70 to 2.87] versus 0.47 [95% CI, 0.00 to 1.24] STRDs/called genome).

Across all three measures, SNVs, STRDs, and LVs, there was no consistent evidence that models allowing for arrested evolution, e.g., in a spore state, in a proportion of cases were a better fit to the data. For SNVs, there was marginal evidence for a better model fit allowing for arrested evolution, and there was stronger support for this model with STRDs; however, for LVs, the model without any arrested evolution was the best fit (Table 1).

**Fig 2:**
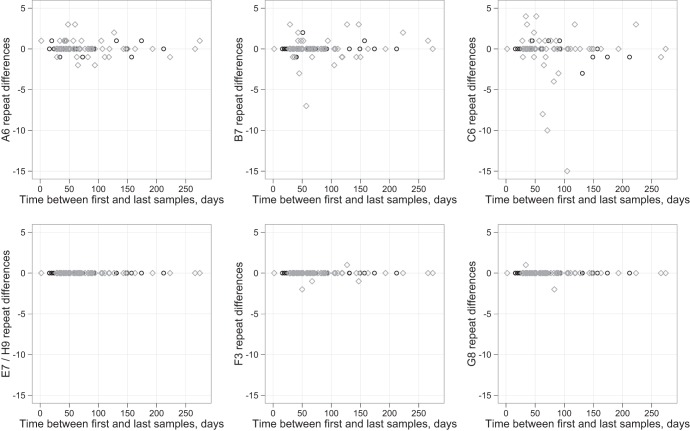
Tandem-repeat differences over time by MLVA locus. Shown are samples from 60 patients with CDI (gray diamonds) and 17 infant asymptomatic carriage episodes (black circles). A single pair of samples separated by 561 days with +1 A6, +1 B7, and +2 C6 repeats was not plotted for ease of visualization. No repeat changes were observed in locus E7 or H9.

**Fig 3:**
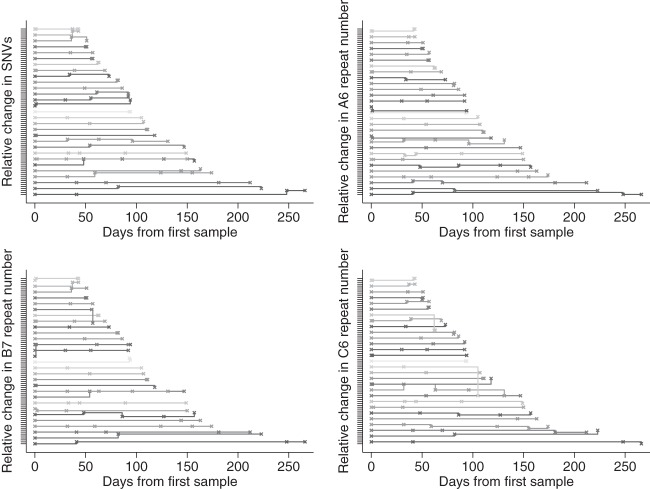
Relative changes in SNV (top left), A6 (top right), B7 (bottom left), and C6 (bottom right) repeat numbers in 29 adults and children sampled more than twice. Samples from the same host are plotted as a horizontal line. Each sample is shown with a cross. The initial vertical position of each line is arbitrary, and vertical deviations of the lines represent changes in the numbers of SNVs or repeats. Each marking on the *y* axis represents a single SNV or repeat.

The A6, B7, and C6 MLVA loci varied the most over time, with no changes seen in E7 or H9 repeat numbers between first and last samples from each host (Fig. 2). At locus C6, there appeared to a slight asymmetry in the changes in tandem-repeat numbers, with large decreases being more common than large increases. In 29 adults and children sampled more than twice (excluding the ribotype 027 reinfection described above), there were multiple instances of change followed by reversion in SNVs and in the A6, B7, and C6 loci (Fig. 3), which is consistent with within-host variation, assay variation, or back mutation. Most changes were ±1 repeat or SNV, although several larger changes in MLVA loci were seen.

**Fig 4:**
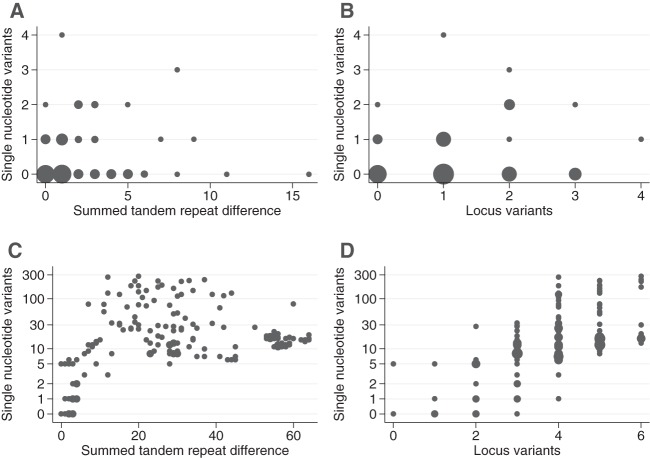
Correlation between single nucleotide variants and summed tandem-repeat differences and between single nucleotide variants and locus variants. The analysis was based on serial samples obtained from 61 symptomatic adults for ongoing or recurrent CDI and 17 asymptomatic carriage episodes in children. (A and B) Relationships for samples obtained from the same host. (C and D) Relationships comparing first samples across different hosts sharing the same ribotype. The *y* axis is plotted using a logarithmic scale. The markers are weighted by the number of samples at each point.

Within the serial samples, there was minimal correlation between SNVs and STRDs, (Spearman's rho = 0.10, *P* = 0.40) and between SNVs and LVs (rho = 0.10, *P* = 0.38); however, many of the serial sample pairs had zero SNVs between them (Fig. 4). When the first samples from each host across different hosts sharing the same ribotype were compared, such that the numbers of SNVs, STRDs, and LVs observed were greater than those within the same host, there was still a limited correlation between SNVs and STRDs (rho = 0.22, *P* = 0.005) but a moderate correlation between SNVs and LVs (rho = 0.53, *P* < 0.001).

### Relative discriminatory powers across serial samples.

The first sample from each CDI case and the first sample from each C. difficile carrier were considered in the pairwise comparisons in order to assess the relative discriminatory powers of MLVA and WGS across the Oxfordshire carriage and disease isolates. Isolates with >2 SNVs (19) and >10 STRDs (5, 13, 14) have been used to exclude direct transmission. Across pairs of samples sharing the same ribotype, 192/283 (68%) were >10 STRDs apart compared with 217/283 (77%) that were different by >2 SNVs. Without prior conditioning on ribotype, 2,898/3,003 (97%) pairs were distinct on MLVA compared with 2,936/3,003 (98%) using WGS.

### Performance for investigating outbreaks.

Sixty-one same-ribotype potential outbreaks involving 300 patients from 31 different hospitals in the United Kingdom were investigated using MLVA and WGS in parallel. Initially, all pairs of patients within each outbreak were compared. As above, transmission between pairs of cases was excluded by WGS and MLVA where pairs of cases were separated by >2 SNVs or >10 STRDs, respectively. Overall, 1,190/1,488 (80%) pairs of patients had concordant results on MLVA and WGS, with transmission possible between 945 (64%) pairs and excluded between 245 (16%) pairs (Table 2). Among the pairs of cases found to be consistent with transmission according to the two measures, there were pairs that were indistinguishable by WGS while showing differences by MLVA, and vice versa (Table 3).

**Table 2:** Classification of 1,488 pairs of same-ribotype cases across 61 potential CDI outbreaks in the United Kingdom^*a*^

No. of SNVs (WGS transmission result)	No. of STRDs (MLVA transmission result)	
	≤10 (transmission possible)	>10 (transmission excluded)
≤2 (transmission possible)	945 (64), 1 (0–2)	69 (5), 3 (2–4)
>2 (transmission excluded)	229 (15), 3 (3–4)	245 (16), 4 (4–5)

a Each cell contains the number of pairs and the percentage of the total number of pairs, followed by the median number (IQR) of LVs in each cell.

**Table 3:** Classification of 945 pairs of same-ribotype cases within 2 SNVs and 10 STRDs across 61 potential CDI outbreaks in the United Kingdom^*a*^

No. of SNVs (WGS result)	No. of STRDs (MLVA result)	
	≤2 (indistinguishable)	3–10 (highly related)
0 (indistinguishable)	395 (27), 1 (0–1)	108 (7), 3 (2–3)
1–2 (highly related)	212 (14), 1 (0–2)	230 (15), 3 (2–3)

a Pairs considered to be consistent with transmission (≤2 SNVs, ≤10 STRDs) were subdivided by whether or not the pairs were considered indistinguishable (0 SNVs, 0 to 2 STRDs) (% of all isolates). Each cell contains the number of pairs and the percentage of the total number of pairs, followed by the median number (IQR) of LVs in each cell.

**Fig 5:**
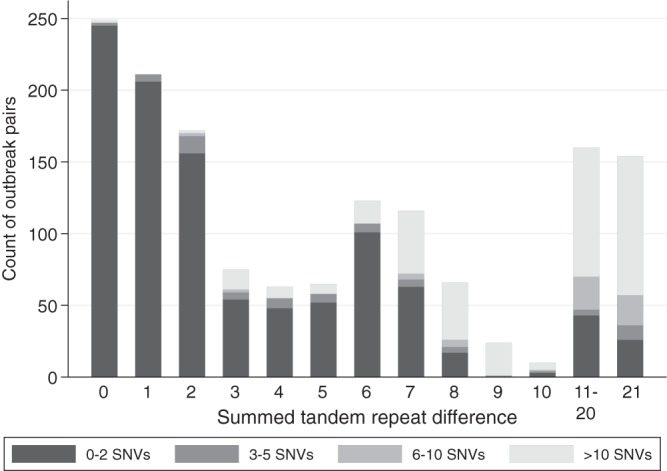
Relationship between STRD and WGS transmission classification. Isolates with ≤2 STRDs are regarded as indistinguishable, and isolates with ≤10 STRDs and ≤2 SNVs have been described as consistent with transmission.

However, 229 (15%) pairs had >2 SNVs between them but ≤10 STRDs, therefore excluding transmission on the basis of WGS cutoffs, where transmission was considered plausible with the use of MLVA thresholds. Similarly, 69 (5%) pairs were found to be related by WGS but were distinct by MLVA. Within the pairs with discordant transmission classifications, there were multiple instances of pairs of cases >2 SNVs apart but that had relatively few STRDs between them, including as few as 2 or 3 STRDs, i.e., that were within the STRD diversity expected from repeated testing of the same host at a given point in time from the models fitted above. Similarly, there were instances of ≤2 SNVs when >20 STRDs were present (Fig. 5).

In pairs that were ≤10 STRDs apart, the median numbers of (IQR) LVs between pairs ≤2 versus >2 SNVs apart were 1 (0 to 2) and 3 (3 to 4), respectively (rank sum *P* = 0.001) (Table 2). This suggests that the extra discrimination from higher numbers of SNVs with few STRDs might be genuine, since the multiple LVs are consistent with the evolution seen in SNVs, despite the changes in repeat number at each locus that did not summate sufficiently to reach the STRD threshold. Similarly, pairs ≤2 SNVs apart with >10 STRDs also had higher numbers of LVs, with a median (IQR) 3 (2 to 4), versus pairs with ≤10 STRDs (*P* = 0.001), suggesting that these are not just large changes in repeat numbers at a single locus but may also reflect genuine discrimination from higher numbers of STRDs with low numbers of SNVs.

### Outbreak classification based on WGS and MLVA.

We also investigated whether the conclusion reached in each potential outbreak investigation based on MLVA would be changed using WGS. Each of the 61 potential outbreaks was classified as (i) all cases belonging to a single outbreak, (ii) all cases being unrelated, or (iii) a mix of related and unrelated cases (5). The classifications were concordant in 58/61 (95%) outbreaks (Table 4). Each of the 3 discordant classifications was explained by a discordant finding in only one case in the investigation. In two cases, WGS appeared to provide the more plausible classification, while in the third, the MLVA result appeared to be more plausible.

**Table 4:** Comparison of outbreak classification using WGS and MLVA

WGS classification	MLVA classification		
	Single outbreak	No transmission	Mix
Single outbreak	33^*a*^	0	1^*b*^
No transmission	0	10^*a*^	1^*c*^
Mix	1^*d*^	0	15^*a*^

a Concordant findings. Each of the 3 discordant classifications was explained by a discordant finding in only one case in the investigation. See footnotes *b* to *d* for explanations.

b Ribotype 106 (12 cases), single case of >10 STRDs apart from closest case, with 13 STRDs, 3 LVs, and 2 SNVs, with all other cases within 7 STRDs and 2 SNVs.

c Ribotype 015 (7 cases), single pair of cases with ≤10 STRDs, 4 STRDs, 3 LVs, and 12 SNVs, with all other cases >10 STRDs from all others, all cases ≥4 SNVs apart.

d Ribotype 027 (3 cases), one case 16 SNVs distinct to next closest case, but 10 STRDs (and 4 LVs) different, all other pairs of cases within 0 SNVs and 4 STRDs.

## DISCUSSION

In this study, we have shown that despite the fact that completely separate regions of the bacterial genome were targeted, the conclusions reached in outbreak investigations using MLVA and WGS were largely concordant. Although WGS offers slightly more discrimination overall, there were also instances where MLVA differentiated between cases that were shown to be highly related using WGS.

Both STRDs or LVs and SNVs evolve relatively slowly, such that most of the variation observed over short time periods is dominated by pre-existing within-host diversity and assay variation; i.e., the numbers of evolutionary events are low. Over short time periods, the stochastic nature of the changes seen in SNVs and STRDs or LVs is reflected in the relatively low correlation seen between these measures in serially sampled individuals. The thresholds for excluding transmission need to account for stochastic evolutionary events; for example, based on a 95% prediction interval, up to 2 SNVs may be consistent with transmission with a zero time difference, despite the expected number of SNVs being as low as 0.29. Given the relatively low rates of evolution compared with onward transmission times, genetic data may need to be combined with epidemiological data and/or probabilistic models in order to accurately reconstruct individual transmission chains. Over longer periods of evolution, such as in comparisons of samples from hosts that are not related by direct transmission (Fig. 4C and D), more evolutionary events occur, lessening the impact of individual stochastic events, and this is reflected in the moderate correlation observed between SNVs and LVs over longer time periods.

Interestingly, although relatively few serial samples from asymptomatically colonized children were included and therefore the power to detect those differences with samples from adults is limited, changes in SNVs and LVs were similar to those from adults with ongoing or recurrent CDI. This suggests that the estimates of within-host diversity and evolution from this study are likely to be generalizable. Although the best-fitting models suggested there might be less STRD within-host diversity in asymptomatic children, they estimated the rate of STRD evolution to be higher than that in adults. Given the relatively small sample size, small improvements in model fit, and the fact that this was not seen for SNVs and LVs, the importance of this finding is unclear.

If WGS is used to exclude transmission, it is important to consider recombination. In our data set, it was uncommon for pairs of samples > 2 SNVs apart, including all identified variants, to be ≤2 SNVs apart after adjustment for recombination; this occurred once in a serial sample pair and once in a transmission cluster. However, if this is not accounted for, transmission may be falsely excluded. As MLVA loci occur at sites that are widely distributed throughout the genome (9), changes at multiple loci are highly unlikely to be introduced by a single recombination event.

Both MLVA and WGS methods require specific expertise. Laboratory experience is required to generate reproducible MLVA results, which may require specific conditions for some PCR ribotypes, and also to generate consistent WGS output. Both techniques also require specific software for analysis; at present, end-to-end commercial solutions for MLVA data are available, whereas sequence data are processed on research pipelines. However, the analysis of data from WGS is likely to become increasingly automated (30). WGS also offers additional benefits, including the reconstruction of long-term evolutionary histories and *in silico* determination of virulence factors and antimicrobial resistance (30). The reagent cost for MLVA in this study was $42 compared to $65 for WGS. MLVA and WGS required similar amounts of hands-on time, with ∼16 h of hands-on time to process 96 samples, which equates to ∼10 min per sample.

The main limitation of this work and similar studies (15) is in the efforts to establish which serially sampled individuals are consistently infected with the same single strain as opposed to being reinfected with a distinct strain. By prior conditioning on ribotyping in this study, clear reinfections were removed. However, reinfections with the same ribotype are still possible but likely constitute only a minority of our cohort. Similarly, although the potential outbreak cases were closely related in time and space, the exact chain of transmission is unknown.

In summary, the MLVA and WGS methods offer broadly similar enhanced discrimination over other genotyping methods in outbreak investigations, and they offer similar per sample costs and laboratory time requirements (according to current prices and technology). Of note, we were unable to predict the MLVA loci from the WGS included here, a consequence of the length of the repetitive regions (up to 306 bp) compared with reads of 100 bp. As WGS read lengths continue to increase, it will become possible to perform *in silico* MLVA on the current MLVA loci and across other repetitive regions. It is therefore likely that WGS will soon be able to exploit both variations in repeat numbers and single nucleotide variants, allowing for the benefits of MLVA and current WGS methods to be delivered by a single platform.
